# The Potential Clinical Utility of Circulating Tumor DNA in Esophageal Adenocarcinoma: From Early Detection to Therapy

**DOI:** 10.3389/fonc.2018.00610

**Published:** 2018-12-11

**Authors:** Juliann E. Kosovec, Ali H. Zaidi, Tamar S. Pounardjian, Blair A. Jobe

**Affiliations:** Esophageal and Lung Institute, Allegheny Health Network, Pittsburgh, PA, United States

**Keywords:** esophageal cancer (EC), circulating tumor DNA (ctDNA), targeted therapy (TT), immunotherapy, personalized medicine

## Abstract

Esophageal adenocarcinoma (EAC) is a lethal cancer requiring improved screening strategies and treatment options due to poor detection methods, aggressive progression, and therapeutic resistance. Emerging circulating tumor DNA (ctDNA) technologies may offer a unique non-invasive strategy to better characterize the highly heterogeneous cancer and more clearly establish the genetic modulations leading to disease progression. The presented review describes the potential advantages of ctDNA methodologies as compared to current clinical strategies to improve clinical detection, enhance disease surveillance, evaluate prognosis, and personalize treatment. Specifically, we describe the ctDNA-targetable genetic markers of prognostic significance to stratify patients into risk of progression from benign to malignant disease and potentially offer cost-effective screening of established cancer. We also describe the application of ctDNA to more effectively characterize the heterogeneity and particular mutagenic resistance mechanisms in real-time to improve prognosis and therapeutic monitoring strategies. Lastly, we discuss the inconsistent clinical responses to currently approved therapies for EAC and the role of ctDNA to explore the dynamic regulation of novel targeted and immunotherapies to personalize therapy and improve patient outcomes. Although there are clear limitations of ctDNA technologies for immediate clinical deployment, this review presents the prospective role of such applications to potentially overcome many of the notable hurdles to treating EAC patients. A deeper understanding of complex EAC tumor biology may result in the progress toward improved clinical outcomes.

## Introduction

Esophageal cancer (EC) is currently the 7th deadliest cancer in the United States with an estimated 17,290 newly diagnosed cases and 15,850 deaths in 2018, with an overall 5-years survival rate under 20% (1). For locally advanced disease, standard of care treatment currently includes neoadjuvant chemoradiotherapy plus surgery (2–4). However, ~40% of patients present with unresectable late-stage disease at diagnosis, reducing their 5-years survival to < 5%, and further underscoring the urgent need for earlier detection and improved treatment strategies (5, 6).

Circulating tumor DNA (ctDNA) is fragmented tumor-derived DNA in the blood stream that serves as a non-invasive diagnostic and prognostic tool for a number of cancer types (7, 8). Current standard cancer liquid protein biomarkers, such as PSA, CA 125, CEA, CA 19-9, AFP, etc., are limited by poor specificity and are known to be elevated in a variety of benign conditions. In contrast, ctDNA offers increased specificity of dysregulated genetic tumor markers, with levels of detection correlating well with premalignant to malignant progression (9). Moreover, ctDNA has a shorter half-life than protein biomarkers, and the real-time information that may be gathered from such “liquid biopsies” may provide unique insight to improve screening methodologies, therapeutic monitoring, and personalized therapy to improve outcomes in a cost-effective widely accessible manner (7, 10). Recently, Park et al. demonstrated that deep sequencing of ctDNA KRAS mutations sensitively detects pancreatic ductal adenocarcinoma and correlates with therapeutic response and disease progression (11). Additionally, ctDNA has shown promise for the detection of post-surgical recurrence of colon, breast, and lung cancers (12–14). The Cancer Genome Atlas (TCGA) recently characterized numerous deregulated genes in esophagogastric adenocarcinoma such as TP53, CDKN2A, SMAD4, ARID1A, ERBB2, VEGFA, CCNE1, GATA4, and GATA6 (15). Many of the identified markers overlapped with significant genes in gastric cancer pathogenesis; however, EAC DNA was consistently more hyper-methylated (11). ctDNA technologies may differentiate these important variances through the detection of not only point mutations, but also copy number variations, chromosomal rearrangements, epigenetic alterations, insertions, and deletions (10). The purpose of this review is to describe the potential utility of ctDNA to improve the detection, monitoring, treatment strategies, and prognosis for EAC patients.

## Literature Review Methods

A thorough literature review was performed by searching the PubMed database for all relevant articles through September 1st, 2018. The following search criteria were applied: (“circulating tumor DNA” OR “ctDNA” OR “cell free DNA” OR “tumor DNA”) AND (“esophageal” OR “esophageal” OR “gastrointestinal” or “gastroesophageal”) AND (“cancer” OR “tumor” OR “malignancy”). The search produced 165 items, and two independent reviewers (JK and TP) screened the results. References from relevant articles were screened as an additional source of literature. Exclusions included articles that evaluated GI malignancies other than esophageal cancer and articles that were not in English. Additional specific searches of current EAC epidemiology statistics, TCGA data, ctDNA methodologies, and EAC therapeutics were performed to supplement background information.

## ctDNA Methodology

ctDNA detection was first established though Sanger sequencing, however limitations such as high cost and complicated protocols have lead to the development of various methodologies to improve efficiency, cost-effectiveness, sensitivity, and specificity (16). Currently, the two main methods of detection include Digital PCR (dPCR) and Next-Generation Sequencing (NGS) (17). dPCR methods, such as Droplet Digital PCR and BEAMing, carry the advantage of being relatively easy, inexpensive methods with short turn around times, which can be a critical advantage when treating aggressive malignancies (18, 19). Additionally, these PCR methods offer superior precision, sensitivity, and reproducibility (19). Specifically, BEAMing has reported up to 100% sensitivity of multiple markers in a variety of cancers (20–22). Although dPCR methods carry higher sensitivity than NGS, it can only detect mutations within a limited number of loci, usually within a single gene, a disadvantage that can be overcome with NGS (23, 24). ctDNA NGS protocols include tagged-amplicon deep sequencing (Tam-Seq), Safe-Sequencing System (Safe-SeqS), and capture based sequencing (CAPP-seq) (23). CAPP-seq targets only areas of interest and is therefore more cost effective and focused than whole exome or whole genome sequencing (16). It also confers the lowest detection limit and background error rate of any NGS-based method and is therefore considered a superior NGS method for practical implementation (16).

Application of ctDNA technologies offers potential unique advantages to clinical care over tissue biopsy, and the concordance of ctDNA with tissue biopsy has been validated across a variety of cancers (25–29). Primarily, tissue DNA collection requires invasive procedures that many frail and elderly patients may not tolerate well (30). Less than 15% of esophageal cancers are diagnosed before the age of 55, so non-invasive detection and monitoring methods may confer particular benefit to the EAC patient population (1). Additionally, it has been well-established that many cancers, including EAC, are very heterogenic in nature and gain additional mutations throughout progression, treatment, and metastasis (31). Tissue biopsy samples only a small section of primary tumor, so obtained samples may not truly reflect all relevant and targetable mutations (32). Comparatively, ctDNA offers a more global perspective of the entire tumor, including metastases, that may offer improved detection of spatial and temporal tumor heterogeneity, which can carry great value by providing up-to-date information on tumor evolution and mutational status throughout disease course (10, 33). Moreover, unlike tissue DNA, analyzing ctDNA does not require fixation of the sample, which can fragment the DNA and cause sequence artifacts that may be misinterpreted as cancer-associated mutations (34).

Despite the improving technologies, there are current limitations to the clinical applications of ctDNA. First, the use of ctDNA as a reliable mechanism to inform clinical decisions lacks standardization due to limited early data and complex bioinformatics processing (35). To date, the only FDA approved tests include methylation-based test of *SEPT9* for colorectal cancer and qPCR-based test for EGFR in non-small cell lung cancer (36, 37). Second, the yield of ctDNA material from plasma is usually quite low, especially in early stage cancers and precursor lesions. In such cases, whole genome amplification has been utilized to improve sample yield, however, future studies are needed to determine if this may compromise the clinical characterization of the tumor (35). Third, the mechanisms and rate of elimination of ctDNA from the bloodstream has not yet been fully explored. There is evidence to suggest ctDNA is cleared through the kidney, liver, and spleen, as well as nuclease degradation and phagocytosis; however, the rate of elimination may be very relevant to elucidate to in the setting of therapeutic monitoring or disease progression (38, 39).

## ctDNA Detection of EAC

In an at-risk patient population of over 20 million GERD sufferers, it is estimated that only 10–15% develop Barrett's esophagus (BE), 0.5% of patients with BE progress to EAC per year, and 25% of high-grade dysplasia cases progress to EAC (40). The minimal risk of disease progression based on GERD symptoms alone dilutes the practicality of universal esophageal cancer screening protocols. Current testing for EAC and precursor lesions is primarily triggered by persistent GERD symptoms in a goal to detect early disease before development to malignancy. Despite this strategy, ~93% of diagnosed EAC patients received no prior surveillance (41). Non-invasive screening with ctDNA liquid biopsy may offer a cost-effective non-invasive alternative to identify patients at increased risk for early-stage disease or development of EAC.

Numerous genetic alterations have been identified in association with the development and prognosis of EAC that may serve as ideal ctDNA targets for improved detection. Particularly, the tumor suppressors TP53 and CDKN2A are mutated in ~72 and 12% of EAC cases, respectively (42, 43). Bettegowda et al. utilized ctDNA technology to detect multiple early and late stage malignancies, including gastroesophageal cancer (GEC), and were able to reliably detect ~58% of localized GEC (no evidence of metastasis) and 100% of metastatic disease (44). As expected, sensitivity improved from Stage 1 through IV progression across all malignancies (44). Additionally, a meta-analysis by Creemers et al. suggested ctDNA detection of HER2 and MYC may be useful for diagnosis and therapeutic monitoring of GEC (45). In order to improve early detection of EAC, non-invasive ctDNA testing may be strategically applied to patients with clinical risk factors, such as recurrent GERD symptoms, Caucasian ethnicity, obesity, smoking history, age, and/or male sex.

Many of the described mutations that characterize EAC, such as TP53 and CDKN2A, also occur in precursor lesions such as BE and HGD, lowering the specificity of such testing for established cancer (46). Still, alternative ctDNA markers may be useful in identifying patients with BE or HGD who are at increased risk for progression to EAC. Currently, the American College of Gastroenterology recommends patients with established non-dysplastic BE should receive endoscopic biopsy surveillance to assess possible disease progression every 3–5 years; however, 90–95% of these patients have completely stable disease and will never progress to EAC (47–50). In a recent study by Li et al. patients with BE were stratified into 79 progressors and 169 non-progressors to EAC, and biopsies were classified based on genetic expression. The study demonstrated that non-progressing BE lesions had small localized deletions at fragile sites, such as FHIT, WWOX, CDKN2A, and 9p arm loss/copy neutral loss of heterozygosity (LOH) (51). These samples revealed a low level of genetic heterogeneity and remained stable over years of surveillance. Alternatively, lesions that progressed to EAC developed increasing chromosome instability as early as 48 months before progression to EAC with gains and losses of whole chromosomes or chromosome arms, such as loss of 18q (51). Progressors showed significant mutation of SMAD4 and were universally more heterogenetic with progressive diversity and genome doublings (51). Similarly, a high-powered meta-analysis by Gharahkahni et al. suggests HTR3C and ABCC5 may be specific for progression of BE to EAC (52). Rumiato et al. recently utilized circulating cell-free DNA (cfDNA) to evaluate the neoplastic progression of BE and were able to successfully detect notable LOH predicting progression to dysplasia and/or EAC prior to visualization (9, 53). Moreover, post-intervention cfDNA sampling demonstrated a return to baseline levels of expression, further validating the potential of the technology to aid in the evaluation of treatment efficacy (9). Therefore, ctDNA screening for SMAD4, HTR3C, ABCC5, and increasing genetic variability over time may stratify patients with precancerous lesions for more appropriate endoscopic screening or intervention recommendations.

## Prognostic Significance and Therapeutic Monitoring

Various previous studies have established ctDNA as a useful tool for the prediction of patient prognosis before and after therapy in a variety of cancer types (54–58). In a recent study evaluating the role of ctDNA for tumor prognosis in breast cancer patients with multiline resistance, Hu et al. revealed unique mutation frequency patterns in those with progression free survival (PFS) <3 months vs. >3 months (59). Additionally, a TP53+PIK3CA mutation pattern successfully predicted progression within 6 months (59). Similarly, in a study investigating the utility of ctDNA to monitor non-small cell lung cancer (NSCLC) patients, tumor and blood samples before and after surgical resection demonstrated that the presence of ctDNA had a higher positive predictive value than six currently utilized clinical tumor biomarkers (60). As EAC is a notably heterozygous malignancy, a variety of diverse studies have reported unique genomic signatures associated with survival and prognostic response to chemoradiotherapy (Table 1) (71). Although, the true clinical utility of these specific genomic profiles has yet to be reliably established, ctDNA may offer an ideal setting for future studies to validate these prognostic indicators, as has been done for other cancers.

**Table 1:** Esophageal adenocarcinoma genetic signatures associated with prognosis.

**Study**	**Signature**	**Results**
Peters et al. (61)	Downregulated: DCK, PAPSS2, SIRT2 Upregulated: TRIM44	Reduction in survival from 58 to 14%
Kim et al. (62)	SPARC, SPP1	Significant association with poor survival
Goh et al. (63)	EGFR, MTMR9, NEIL2, WT1	Stratification of patients in 5 survival clusters
Pennathur et al. (64)	165-gene signature	Stratification of patients into good vs. poor survival cluster
Rao et al. (65)	59-gene signature	Stratification of patients into good vs. poor survival cluster
Rao et al. (65)	Upregulated: Ephrin B3	Increased response to chemoradiotherapy
Motoori et al. (66)	Downregulated: PERP Upregulated: DAD1, PRDX6, SELPINB6, and SRF	Decreased response to chemoradiotherapy
Maher et al. (67)	Downregulated: ERB41L3, NMES1, RPNC1, STAT5B Upregulated: RTKN	Increased response to chemoradiotherapy in EAC and ESCC
Tamoto et al. (68)	Upregulated: PERP, S100A2, SPRR3	Characterized complete responders to chemoradiotherapy in EAC and ESCC
Murugasu et al. (69)	Increased tumor heterogeneity	Decreased response to neoadjuvant chemotherapy
Rumiato et al. (70)	SNPs of ABCC2, ABCC3, CYP2A6, PPARG, SLC7A8	Decreased response to platinum-based chemotherapy

Furthermore, 44–61% of patients treated according to the current guidelines of neoadjuvant chemoradiotherapy plus surgery will experience recurrent disease (72). Despite complete resection with pathologically-confirmed clear margins and negative post-therapeutic CT imaging, it is hypothesized that many EAC recurrences are due to minimal residual disease (MRD) or systemic micrometastases (73). Previous studies have demonstrated that ctDNA screening may more sensitively identify small areas of remaining or recurrent disease as compared to standard imaging technology (74, 75). Such sensitive screening methodology may be ideally suited for such an aggressively deadly disease to trigger early intervention and reduce associated mortality. Recently, Chan et al. was able to improve detection of nasopharyngeal carcinoma recurrence by 10 months as compared to standard screening protocols (76). Similarly, ctDNA has also been used to detect MRD and predict recurrence in breast cancer with a mean lead-time of 7.9 months over clinical relapse (12). In a third study for lung cancer, ctDNA mutations predicted recurrence with 94% sensitivity with a median clinical lead-time of 5.2 months (14).

Unfortunately, 50–60% of EAC patients are resistant to standard chemotherapeutic treatment options due to inherent heterogeneity and development of escape mechanisms (69, 77). ctDNA technology provides an additional tool to monitor real-time therapeutic efficacy for more efficient modification of dosing and regimen. Murugasu et al. demonstrated that patients with EAC who had promising response to platinum agents developed decreased C>T mutations and increased C>A mutations through the course of treatment (69). Findlay et al. also confirmed this finding in addition to TT>CT changes, and acquired mutations in SF3B1, TAF1, and CCND2 (78). As neoadjuvant chemotherapy can dramatically and rapidly change the EAC genome profile, real-time monitoring to quickly identify resistance and opportunities for new actionable mutations may lead to clinical benefit. Moreover, ctDNA studies may provide additional insight into disease progression when used in conjunction with clinical imaging. de Figueriredo Barros et al. described an increasing mutation burden correlating with progression in metastatic colorectal cancer, while CT imaging showed stable disease (79).

## Personalized Therapeutic Applications

Various targeted therapies have been explored for the treatment of gastroesophageal cancers but have only demonstrated limited efficacy (80, 81). Only trastuzumab has been established as a potential first-line treatment option for advanced GEC in HER2+ patients; however, benefits are minimal with a median overall survival (OS) of 13.8 months vs. 11.1 months (82). Ramucirumab (VEGFR-2) single agent or in combination with paclitaxel are recommended options for second-line treatment demonstrating an OS of 5.2 and 9.6 months, compared to 3.8 and 7.4 months, respectively (83, 84).

Over the last decade, success of immunotherapy across multiple cancer types has prompted exploration of novel immunologic targets for GECs (85). Recently, the late-line KEYNOTE-059 study of pembrolizumab in PD-L1 positive GEC and gastric tumors showed an objective response rate of 13.3% with 58% of the responses lasting 6 months or longer, leading to a third-line FDA approval (86, 87). Similarly, nivolumab has been approved for third-line GEC treatment in Japan after demonstrating an improved median survival of 5.26 months vs. 4.14 months, independent of PD-L1 expression (88, 89). Disappointingly, the most recent KEYNOTE-061 second-line pembrolizumab trial did not demonstrate any significant survival benefit in PD-L1 positive GEC patients (90). Still, pembrolizumab has secured a tumor agnostic approval in microsatellite instability-high or mismatch repair deficient previously treated unresectable or metastatic solid tumors; yet, these criteria only apply to ~3% of GECs (15, 91).

First generation immuno-oncology agents have demonstrated modest activity and potential application for the treatment of EAC; however, better stratification biomarkers and newer immunotherapeutic combination strategies may be required for enhanced durable responses. Only ~40% of EAC patients present with baseline PD-1 positivity, and this expression occurs primarily at the invasive margin (92, 93). Therefore, PD-L1 may not be the ideal predictive marker in EAC due to the inconsistent clinical response and relatively low rate of upregulation. There is evidence that the immune microenvironment as a whole is highly reactive to chemoradiotherapy and significantly increases expression of PD-L1 and other developing targets, such as LAG3, TIM3, and OX40 (94). Therefore, ctDNA technology may be a useful tool to explore the dynamic immunoregulation throughout the course of treatment and to better characterize the immunologic profile of EAC beyond the PD-1/PD-L1 pathway. A number of novel targeted molecular and immunotherapeutic agents are currently under investigation for EAC to explore additional potential treatment alternatives (95). Due to the extreme genomic variability and instability that classically characterize EAC, in addition to the multiple resistance mechanisms that may emerge during treatment, EAC may be an optimal candidate for the application of personalized therapeutics through the use of real-time ctDNA monitoring (96).

Moreover, in patients treated with immunotherapy, radiological evaluation of early disease responsiveness is especially challenging due to immune cell infiltration resulting in pseudo-progression on imaging (97, 98). Cabel et al. showed ctDNA is useful for early monitoring of responsiveness to anti-PD1 immunotherapy and correlated well with PFS and OS (99). Similarly, Xi et al. showed early changes in BRAFV600E ctDNA as early indicator to identify responding from non-responding patients with metastatic melanoma treated with immunotherapy (100). In another study, Raja et al. demonstrated early reduction in ctDNA correlation with survival in lung and bladder cancer after treatment with durvalumab (PD-L1) (101). Lastly, Khagi et al. demonstrated increased ctDNA detection of variants of unknown significance correlated with statistically significant improved PFS and OS in patients with diverse malignancies receiving immunotherapy (102). Exploration into the use of ctDNA technologies for non-invasive therapeutic monitoring of immunotherapy in EAC may be warranted due to the supporting evidence that ctDNA may be a good indicator of immunoresponse and prognosis.

## Conclusions

Esophageal adenocarcinoma is a very deadly disease due to the high percentage of patients presenting with late-stage diagnosis and minimally effective treatment strategies. The emergence of non-invasive and cost-effective ctDNA technologies may offer a unique opportunity to improve screening protocols to more effectively monitor benign disease and detect malignancy earlier. Moreover, many patients with EAC are resistant to first line chemoradiotherapy, and therapeutic monitoring of ctDNA mutations throughout the course of treatment may allow for more efficient adjustments of personalized therapy. Despite only minor successes with targeted therapies due to the highly heterogenic nature of EAC, real-time information regarding response and prognosis may allow for more informed clinical decision-making strategies. Additionally, the integration of ctDNA with developing immunotherapeutic options may open the door for improved prognostic outcomes.

Limitations of ctDNA technologies are rooted in the still early development of this new emerging technology and the lack of strongly validated studies characterizing the precise clinical role it may play to truly improve patient care (30). Rigorous future clinical studies will be required to reliably describe specific discoverable changes in ctDNA throughout disease progression before broad implementation. Still, EAC remains an extremely lethal disease, and further investigation into the potential benefits of ctDNA characterization may offer significant benefits to improve early detection, monitor progression, enhance real-time personalization of therapeutics, and evaluate prognosis to improve clinical care.

## Author Contributions

This manuscript was drafted by JK, AZ, and TP and was critically reviewed by BJ.

### Conflict of Interest Statement

The authors declare that the research was conducted in the absence of any commercial or financial relationships that could be construed as a potential conflict of interest.
